# Kronecker Product Linear Exponent AR(1) Correlation Structures for Multivariate Repeated Measures

**DOI:** 10.1371/journal.pone.0088864

**Published:** 2014-02-21

**Authors:** Sean L. Simpson, Lloyd J. Edwards, Martin A. Styner, Keith E. Muller

**Affiliations:** 1 Department of Biostatistical Sciences, Wake Forest School of Medicine, Winston-Salem, North Carolina, United States of America; 2 Department of Biostatistics, University of North Carolina at Chapel Hill, Chapel Hill, North Carolina, United States of America; 3 Departments of Psychiatry and Computer Science, University of North Carolina at Chapel Hill, Chapel Hill, North Carolina, United States of America; 4 Department of Health Outcomes and Policy, University of Florida, Gainesville, Florida, United States of America; Institute of Psychology, Chinese Academy of Sciences, China

## Abstract

Longitudinal imaging studies have moved to the forefront of medical research due to their ability to characterize spatio-temporal features of biological structures across the lifespan. Credible models of the correlations in longitudinal imaging require two or more pattern components. Valid inference requires enough flexibility of the correlation model to allow reasonable fidelity to the true pattern. On the other hand, the existence of computable estimates demands a parsimonious parameterization of the correlation structure. For many one-dimensional spatial or temporal arrays, *the linear exponent autoregressive* (LEAR) correlation structure meets these two opposing goals in one model. The LEAR structure is a flexible two-parameter correlation model that applies to situations in which the within-subject correlation decreases exponentially in time or space. It allows for an attenuation or acceleration of the exponential decay rate imposed by the commonly used continuous-time AR(1) structure. We propose the Kronecker product LEAR correlation structure for multivariate repeated measures data in which the correlation between measurements for a given subject is induced by two factors (e.g., spatial and temporal dependence). Excellent analytic and numerical properties make the Kronecker product LEAR model a valuable addition to the suite of parsimonious correlation structures for multivariate repeated measures data. Longitudinal medical imaging data of caudate morphology in schizophrenia illustrates the appeal of the Kronecker product LEAR correlation structure.

## Introduction

Multivariate repeated measures studies are characterized by data that have more than one set of correlated outcomes or repeated factors. Spatio-temporal data fall into this more general category, since the outcome variables repeat in both space and time. Valid analysis requires accurately modeling the correlation pattern. Muller et al. and Gurka et al. showed that under-specifying the correlation structure can severely inflate test size of tests of fixed effects in the general linear mixed model [1], [2]. Modeling the correlation pattern separately for each repeated factor with multivariate repeated measures data has substantial advantages. Most important, the approach allows for the choosing and tuning of each model separately, which improves accuracy and makes model fitting easier. Furthermore, the approach uses fewer parameters than an unstructured model. The Kronecker product combines the factor-specific correlation structures into an overall correlation model.

### Separable Correlation Models

Galecki [3] gave a detailed treatment of Kronecker product covariance structures, also known as separable covariance models. A covariance matrix is *separable* if and only if it can be written as 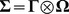, where 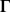 and 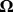 are factor-specific covariance matrices (e.g. the covariance matrices for the temporal and spatial dimensions of spatio-temporal data respectively). A key advantage of the model is the ease of interpretation of the independent contribution of every repeated factor to the overall within-subject error covariance matrix. Galecki, Naik and Rao, and Mitchell et al. [3]–[5] detailed the computational advantages of the Kronecker product covariance structure. The partial derivatives, inverse, and Cholesky decomposition of the overall covariance matrix can be performed more easily on the factor-specific models because they have much smaller dimensions.

While separable covariance models are commonly used in the spatial statistics literature [6], they have rarely been used in multivariate longitudinal (and more generally, multivariate repeated measures) data analysis. To our knowledge, no commonly used statistical packages provide a flexible framework for implementing the structures, limiting their use to those with the appropriate programming skills. For example, SAS version 9.3 [7] has only three Kronecker product covariance structures (unstructured matrix paired with either an unstructured, compound symmetric, or discrete-time AR(1) matrix). Given the advantages of separable models, extending software to allow general implementation is important for researchers in a variety of areas. For example, longitudinal group-randomized controlled trials often have within-group correlations (e.g., by school for youth based studies) and within-subject, longitudinally induced correlations [8]. Such data can be modeled effectively with the Kronecker product of a compound symmetric and LEAR correlation structure.

**Figure 1:**
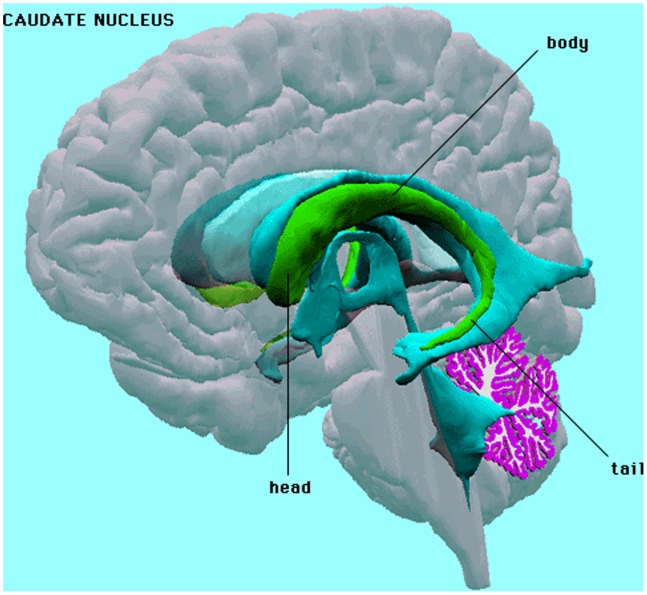
The Caudate Nuclei in the Human Brain.

Brain morphology research is another area in which separable models would be effective because both the shape of brain structures (spatial correlation) and how they change over time (temporal correlation) must be analyzed. Our work concerns temporal changes in caudate morphology in schizophrenics. Schizophrenia is characterized by disabling impairments in the perception or expression of reality. Pathological changes in brain morphology in schizophrenics may be progressive and associated with clinical outcome. Much recent work has focused on the effect of antipsychotic drugs on brain morphology [9], [10]. The drugs were tested on the caudate (shown in **Figure 1**), an important part of the brain’s learning and memory system. Assessing drug efficacy requires proper analysis of temporal shape changes in the caudate, which can be modeled with separable correlation structures.

Limitations of separable models have been noted by various authors. Most important, as mentioned in [11], patterns of interaction among the various factors cannot be modeled when utilizing a Kronecker product structure. Within a given subject, all factors must have *consistently-spaced* measurements. In the context of spatio-temporal data, this means that at each time point a given subject must have the same number of measurements taken at the same spatial locations.

### An Appealing and Flexible Separable Correlation Model

Fitting a Kronecker product structure requires choosing models for each of the factors. In medical imaging, repeated measures dimensions typically have within-subject correlation decreasing exponentially in time or space. The continuous-time, first-order autoregressive correlation structure, denoted AR(1), is often used in longitudinal settings. This model was briefly examined in [13] and is a special case of the model described in [14]. Despite its wide use, the AR(1) structure often poorly gauges within-subject correlations that decay at a slower or faster rate than required by the AR(1) model. The *linear exponent autoregressive* (LEAR) correlation model, defined in **Table 1** (reproduced from [15]) and equations 1 and 2 below, overcomes this limitation by allowing an attenuation or acceleration of the exponential decay rate imposed by the AR(1) structure [15]. **Table 1** also defines the AR(1) model along with other stationary correlation structures that are continuous functions of distance. The focus on stationary models reflects the desire to maintain parsimony across a variety of data types. Moreover, the greater complexity of non-stationary models does not seem necessary for the limited applications of interest. The exponential model defined in Table 1, discussed almost exclusively in the spatial statistics literature, is in fact equivalent to the continuous-time AR(1) model with 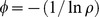. As proposed in [15], we believe that the AR(1) and damped exponential (DE) models serve as the most relevant competitors to the LEAR structure. Special cases of both the LEAR and DE families include the AR(1), compound symmetry, and first-order moving average (MA(1)) correlation structures.

**Table 1:** Stationary Correlations Structures That are Continuous Functions of Distance.

Structure	( 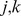 )th element 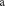 , 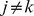	Params	Data Types 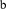
LEAR	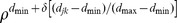	2	L/T,S,O
AR(1)	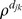	1	L/T,S,O
DE	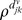	2	L/T,S,O
GAR(1)	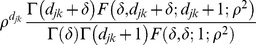	2	L/T
Exponential	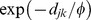	1	S
Gaussian	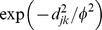	1	S
Linear	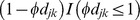	1	S
Matern	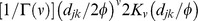	2	S
Spherical	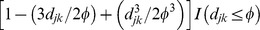	1	S

NOTE: [41] and [42] detail the DE and GAR(1) structures respectively. See [43] for further details regarding the spatial structures.

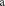
elements of 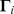 and/or 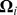
from equation 3.

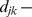
distance between 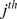 and 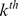 measurement of 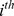 subject.

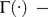
gamma function.

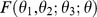
 – hypergeometric function.

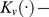
modified Bessel function of the second kind of (real) order 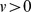.

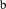
L/T: Longitudinal/Time Series.

S: Spatial.

O: Other.

The advantages of employing a LEAR model for each component led us to consider a Kronecker product LEAR correlation structure for multivariate repeated measures data in which the correlation between measurements for a given subject is induced by two factors. We allow for an imbalance in both dimensions across subjects, i.e., an unequal numbers of observations. The LEAR model also accommodates any arbitrary spacing within a dimension. We use maximum likelihood estimation of the general linear model with Gaussian errors to illustrate the benefits of the structure. All other common estimation methods for linear and nonlinear models could also be used with a Kronecker product LEAR structure.

## Materials and Methods

### Ethics Statement

This analysis involved the application of a new method to extant data. The original study was conducted from March 1, 1997 to July 31, 2001 at 14 academic medical centers. All subjects gave written informed consent in accordance with the Declaration of Helsinki. Each site’s institutional review board (University of North Carolina School of Medicine, Chapel Hill, NC; Emory University School of Medicine, Atlanta, GA; McLean Hospital, Harvard Medical School, Belmont, MA; John Umstead Hospital, Duke University Health System, Butner, NC; University of Florida, Gainesville, FL; Massachusetts Mental Health Center, Harvard Medical School, Boston, MA; University of Massachusetts Medical Center, Worchester, MA; University of Pennsylvania Medical Center, Philadelphia, PA; University of Toronto School of Medicine, Toronto; University of Cincinnati, Cincinnati, OH; Stanford University School of Medicine, Stanford, CA; University of Michigan Medical Center, Ann Arbor, MI; University Hospital Utrecht, Utrecht, the Netherlands; Maudsley Hospital, Institute of Psychiatry, London) approved the study.

### Example Data: Schizophrenia and Caudate Morphology

**Figure 2:**
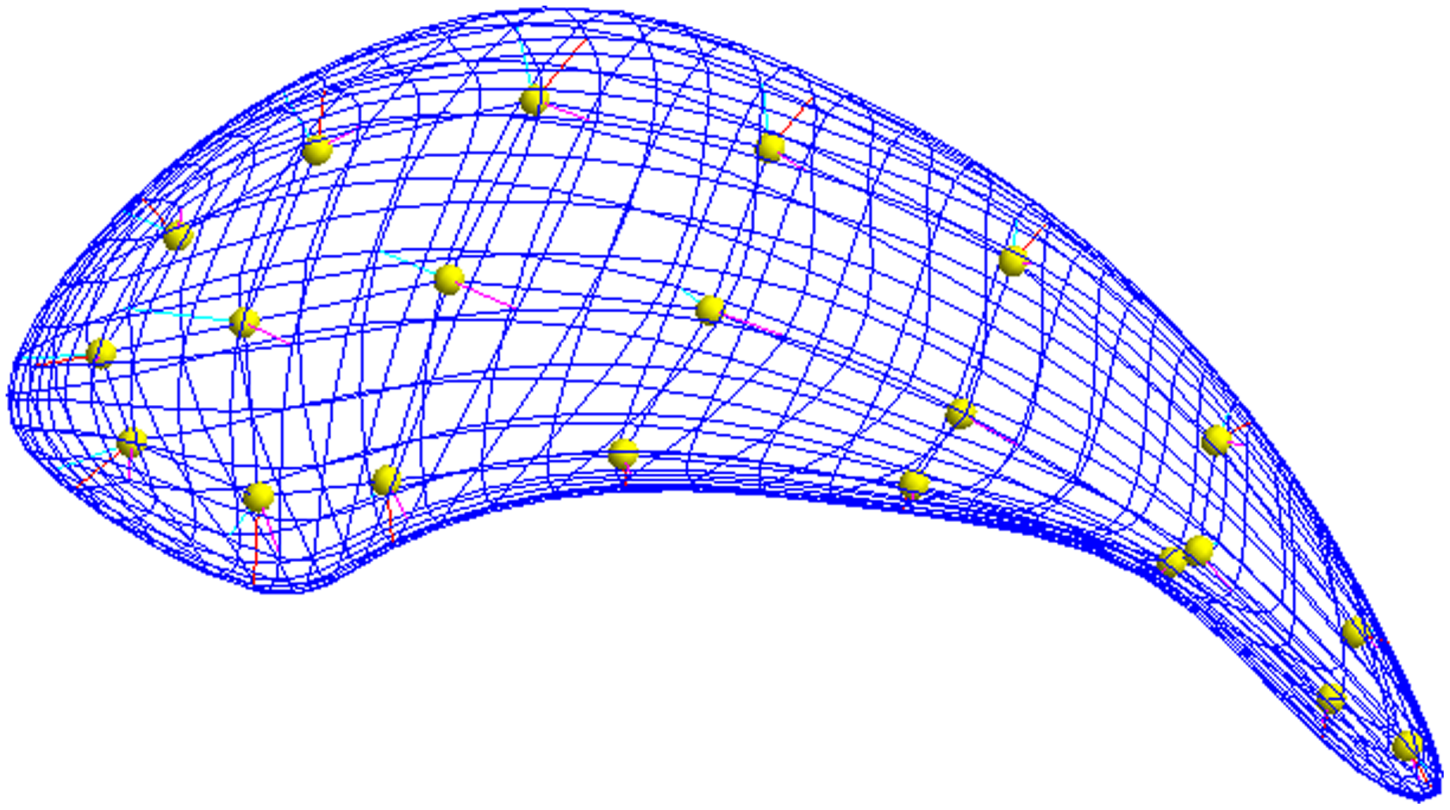
M-rep shape representation model of the caudate.

Our data came from longitudinal MRI scans of the left caudate for 240 schizophrenia patients and 56 controls. The surface of each object was parameterized via the medial representation (m-rep) method as described in [16]. The caudate shape was determined as a 3×7 grid of medial mesh points (spherical nodes) (see **Figure 2**). Data were reduced to one outcome measure: *radius* in cm as a measure of local object width at an m-rep node (21 locations per caudate). This measure is represented in **Figure 2** by the length of the spokes emanating from the spherical nodes to the surface of the object. The distance between two radii for a given subject was calculated as the mean Euclidean distance over all images. The schizophrenia patients were randomized to either haloperidol (a conventional antipsychotic) or olanzapine (an atypical antipsychotic). Scans were taken up to 47 months post-baseline with the median and maximum number of scans per subject being three and seven respectively. The two treatment groups were combined in order to avoid undermining ongoing research, as the final trial results comparing the treatments have not yet been published. The other covariates of interest were age, gender, and race. Preliminary analyses (based on the same test discussed in the Results section, namely the residual approximation of the 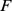-test for a Wald statistic) showed that the shape of the caudate, and thus the radii, differs significantly at baseline between schizophrenics and controls. The study hypothesized that the neuroprotective effect of the drugs would lead to no overall differences in shape between the patients and controls. An example subset of one subject’s data is given in **Table 2**.

**Table 2:** Example subset of one subject’s data (Treatment - Olanzapine, Gender - M, Age - 20).

	log 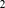 (radius)				
Node #	Baseline	Month 3	Month 12	Month 24	Month 47
1	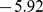	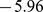	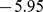	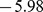	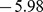
2	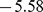	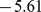	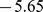	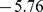	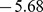
3	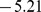	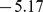	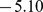	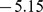	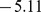
4	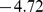	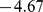	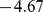	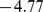	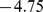
5	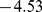	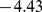	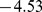	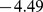	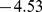
6	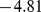	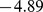	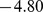	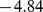	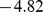
7	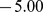	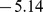	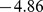	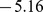	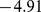
8	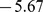	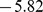	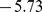	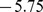	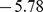
9	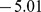	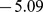	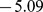	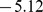	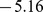
10	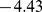	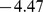	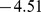	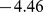	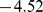
11	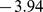	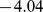	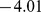	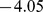	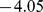
12	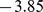	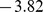	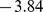	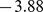	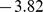
13	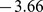	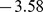	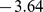	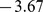	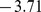
14	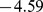	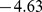	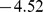	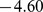	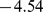
15	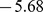	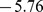	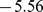	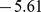	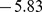
16	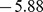	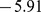	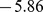	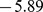	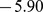
17	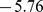	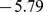	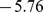	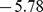	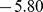
18	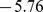	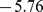	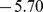	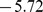	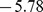
19	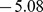	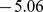	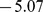	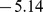	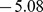
20	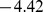	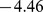	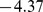	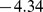	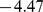
21	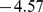	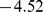	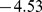	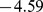	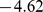

### Model Definition

Suppose 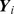 is a 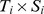 matrix of observations (e.g., 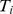 temporal measurements and 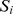 spatial measurements) on the 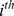 subject 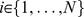. Let 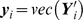 be the 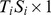 vector of the 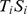 observations. Here, 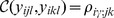 and 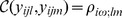 represent the temporal (or factor 1) and spatial (or factor 2) correlations, respectively, for 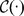 the correlation operator. Then for 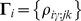 (the temporal/factor 1 correlation matrix) and 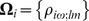 (the spatial/factor 2 correlation matrix), the factor-specific *linear exponent autoregressive* (LEAR) correlation structures are
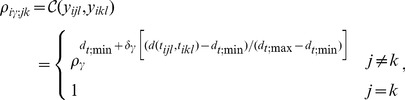
(1)

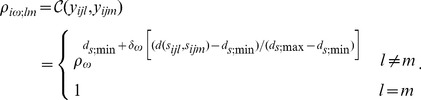
(2)


The Kronecker product LEAR correlation structure is
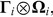
(3)where 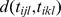 and 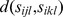 are the distances between measurement times and locations respectively. In turn, 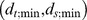 and 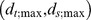 are computational constants equal to the minimum and maximum number of temporal and spatial distance units across all subjects. Parameters 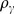 and 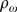 are the correlations between observations separated by one unit of time and distance respectively, and 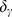 and 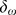 are the decay speeds. We assume 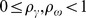 and 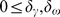. The 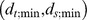 and 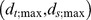 constants allow the model to adapt to the data and scale distance such that the multiplier of the decay speeds 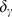 and 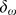, 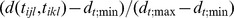 and 
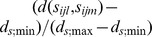
, is between 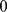 and 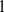 for computational purposes. One could also consider tuning the constants if necessary to address convergence issues. Simpson et al. [15] gave details on setting the distance constants. Ensuring that the factor-specific matrices 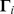 and 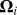 are positive definite (as discussed in [15]) is sufficient for ensuring the positive definiteness of 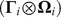 (Theorem 7.10, [17]). Note that each factor-specific LEAR model can also be reparameterized as the Hadamard product of an equal correlation and a continuous-time AR(1) model, as detailed in [15].

**Figure 3:**
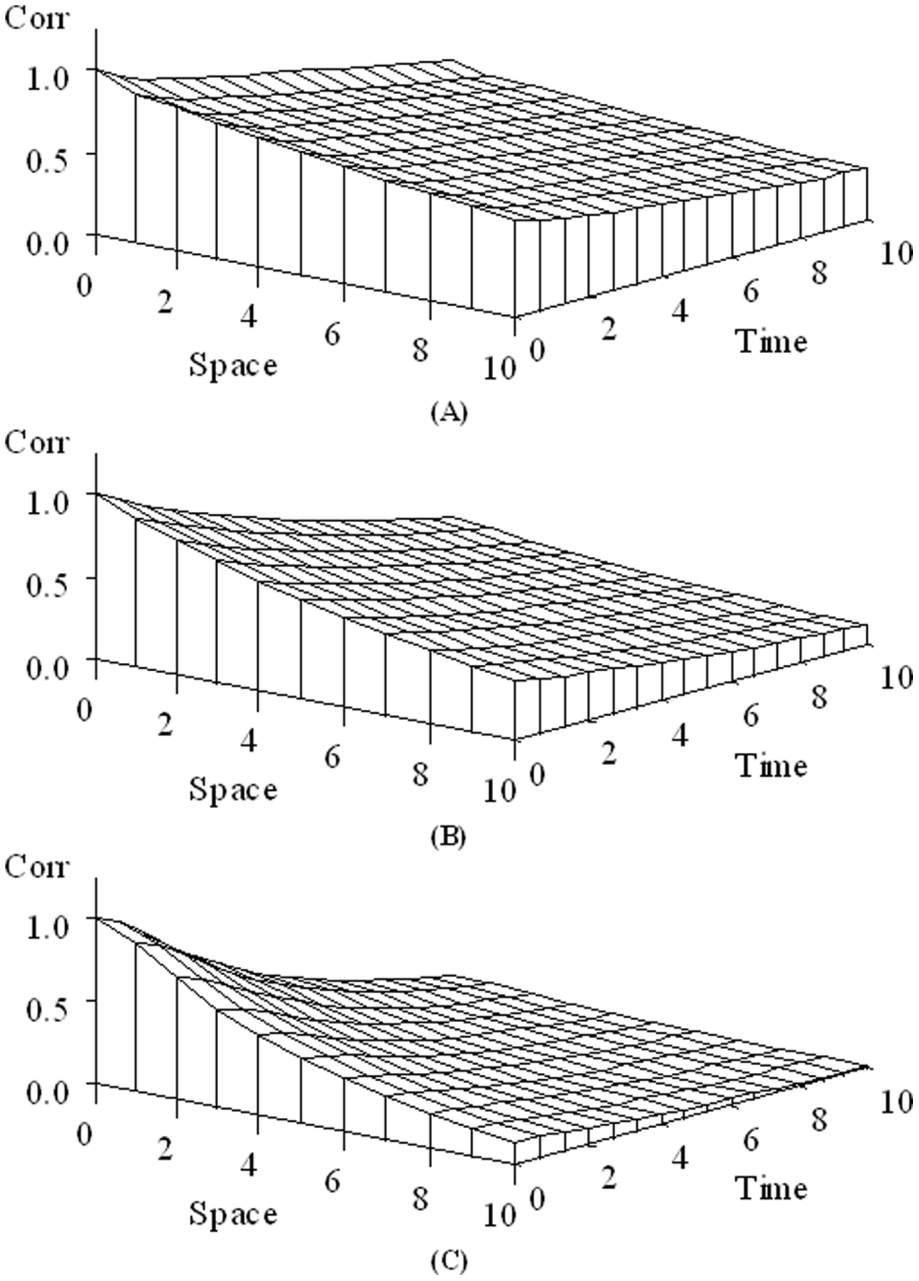
Plot of correlation as a function of spatial and temporal distance. (**A**) Both factor specific matrices have a decay rate that is slower than that of the AR(1) model with correlation parameters 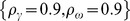 and 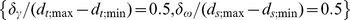. (**B**) Both factor specific matrices have an AR(1) decay rate with correlation parameters 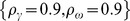
and 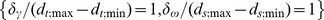. (**C**) Both factor specific matrices have a decay rate that is faster than that of the AR(1) model with correlation parameters 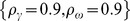 and 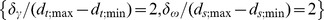.

Graphical depictions of the Kronecker product LEAR structure help to provide insight into the types of correlation patterns that can be modeled. A correlation pattern in which both of the factor-specific matrices (e.g. spatial and temporal matrices) have decay rates slower than that of the AR(1) model is illustrated in **Figure 3A**. **Figures 3B and 3C** exhibit patterns with dual AR(1) and faster than AR(1) decay rates respectively.

Given the advantages of the Kronecker product covariance model, we believe that it has been underutilized in practice. As alluded to in the Introduction, the model has great computational properties and simplifies interpretation. It also reduces the dimension of the calculations, sometimes drastically (e.g., having two 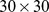 matrices vs. a 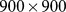 matrix), while allowing complex factor-specific correlation structures. These inherent qualities make the Kronecker product covariance model an appealing and useful tool (among the suite of tools) in many High Dimensional, Low Sample Size contexts common in medical imaging and various kinds of “-omics” data. Modeling the factor-specific matrices with the LEAR structure is especially attractive due to the increased flexibility, parsimony, and numerical stability resulting from this combination.

Here, we adopt the technique of modeling the correlation and variance structures separately as seen in [18] and others. With the assumptions of covariance model separability and homoscedasticity, an equal variance Kronecker product structure has great appeal. The overall within-subject error covariance matrix is then defined as
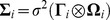
(4)for the 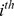 subject or independent sampling unit. The formulation has several advantages. The reduction in the number of parameters leads to computational benefits. The model is also identifiable since 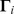 and 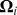 will necessarily be correlation matrices. When heteroscedasticity is present, 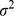 can be thought of as an aggregate variance parameter for the two factors. The robustness of the equal variance model to deviations from homoscedasticity likely depends on the accuracy and flexibility of the specified correlation matrices 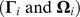 and its examination will be left to future work. We focus on modeling the correlation and assume an equal variance structure for the application of interest.

### Model Estimation

The Kronecker product LEAR structure can be imbedded within various modeling and estimation methods. The best approach may vary by context. With linear structured, factor-specific matrices, the noniterative approach in [19] has appeal. However, the approach is not appropriate for the LEAR structure given its nonlinear nature. Many have used maximum likelihood methods for parameter estimation in a Kronecker product model [4], [5], [12], [20]–[24], but none of their approaches allow for data that are unbalanced in both dimensions. As noted in [25] and others, the Kenward-Roger approach with REML estimation is preferable for small sample estimation and inference. Non- and semiparametric approaches, as used in [18], [26], may prove beneficial for non-Gaussian data or when a nonparametric variance function is appropriate (which could still be coupled with the parametric LEAR correlation functions). However, fully parametric covariance functions (parametric variances and correlations) may provide more informative results and yield more powerful inference. The Kronecker product LEAR model may also serve as a plausible working correlation structure in a generalized estimating equation (GEE) framework. We focus on Gaussian data with moderately large sample sizes, and leave the examination of the Kronecker product model in other contexts to future work.

We consider maximum likelihood (ML) estimation of the general linear model where the Gaussian errors have a Kronecker product LEAR correlation structure. We allow for an imbalance in both dimensions of the data. Our moderately large sample context precludes the need for REML estimation.

Consider the following general linear model for multivariate repeated measures data with the Kronecker product LEAR correlation structure:
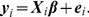
(5)


Again, 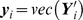, with 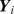 being a 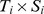 matrix of observations (e.g., 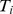 temporal measurements and 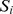 spatial measurements) on the 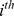 subject 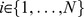. Thus, 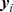 is a 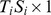 vector of the 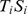 observations, 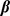 is a 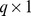 vector of fixed and unknown population parameters, 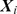 is a 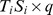 fixed and known design matrix corresponding to the fixed effects, and 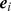 is a 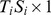 vector of random error terms. We assume 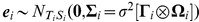 is independent of 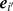 for 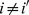, where 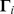 and 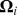 are defined in equations 1 and 2.

Setting 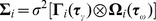, where 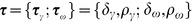, the log-likelihood function of the parameters given the data under the model is:
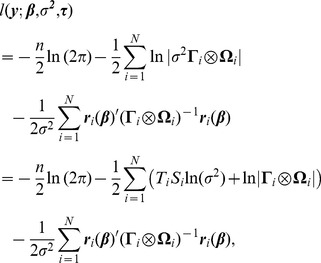
(6)where 
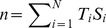
 and 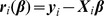. The ML estimates are derived following the approach employed in [15]. After profiling 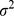 out of the likelihood, the profile log-likelihood is given by



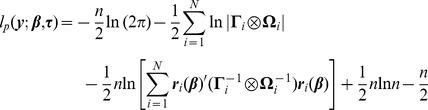
(7)To avoid computational issues it is best to use the equality
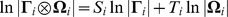
in case 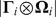 is close to zero.

The ML estimates of the model parameters may be computed with the Newton-Raphson algorithm, which requires the first and second partial derivatives of the profile log-likelihood. The derivations of the first partial derivatives are available from the authors. The second partial derivatives of the parameters, which are employed to determine the asymptotic variance-correlation matrix of the estimators, may be approximated by finite difference formulas. The derivative approximations are detailed in [27], [28]. The 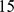 analytic second derivatives can be derived explicitly as in [15]. However, the approximations have proved very accurate.

After getting the estimates of 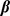 and 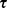 utilizing the Newton-Raphson algorithm, an estimate of 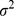 is calculated by substituting the estimates into 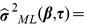 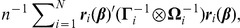 which is the expression resulting from the initial profiling of 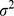 out of the likelihood. An estimator of the variance for 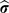 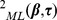, assuming that 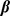 and 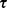 are known, is then
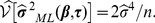
(8)


The derivation of this estimator is available from the authors. A SAS IML [7] program implementing this estimation procedure for the general linear model with a Kronecker product LEAR correlation structure is also available upon request, and a more general macro is in development.

A possible complication when implementing the Kronecker product LEAR correlation model is that the proposed estimation method can produce negative variance estimates for the correlation parameters. This may occur for the parameters of either one or both of the factor-specific matrices when there is a faster decay rate than that imposed by the AR(1) model coupled with a “small” 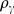 and/or 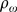. The instability of the second order derivatives of the objective function, resulting from the small, quickly decaying correlation(s), leads to this problem. More specifically, when the log likelihood is shallow, approximately quadratic in the parameter being considered, the second derivatives will be near zero analytically, and therefore indistinguishable from zero numerically, so finite precision arithmetic can lead to negative values with nonzero probability.

An alternate approach is to implement an estimation method which only uses first-order derivatives such as a quasi-Newton procedure. An efficient modification of Powell’s [29]–[32] Variable Metric Constrained WatchDog (VMCWD) algorithm is often used. A quadratic programming subroutine updates and downdates the Cholesky factor, as detailed in [33]. However, quasi-Newton approaches generally have less stability and worse convergence properties than the Newton-Raphson method. Another approach is to recognize this complication as a diagnostic tool. Since a correlation matrix of this nature (one with most off-diagonal elements being close to zero given the small correlation and fast decay rate) is approximately equal to the identity matrix, an independence model may be the best fit for the factor specific structure in this situation.

## Results

We model the caudate data with the general linear model for multivariate repeated measures data defined in the previous section (all modeling assumptions were assessed and met as discussed in [34]). The initial full mean model is as follows:
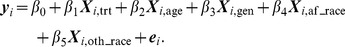
(9)


The log
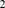
(radius) values for each of the 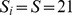 locations (spatial factor) and 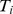 images (temporal factor) for each subject are contained in 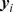 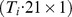. The vectors 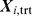, 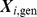, 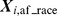 and 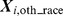 indicate the treatment group (patients and controls), gender, and race (African-American, Other, and White–reference group) of the 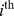 subject respectively. The ages at baseline are contained in 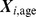.

We first assume a separable covariance and model the temporal and spatial factor-specific correlations of the model errors with continuous-time AR(1), DE, and LEAR structures in order to assess the best model via the AIC 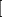
AIC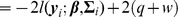, where 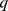 is the number of fixed effect parameters and 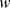 is the number of unique covariance parameters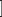. **Table 3** contains the AIC values for all nine possible correlation model fits with the initial full mean model. Modeling both the temporal and spatial correlations with the LEAR structure provides the best model fit of the nine combinations. The BIC 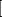
BIC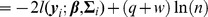, where 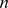 is the total number of observations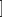 corroborates the differences in fits. The resulting parameter estimates and p-values (based on the residual approximation of the 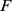-test for a Wald statistic [34]) associated with each of the covariates are presented in **Table 4** for three of the correlation model fits: LEAR
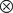
LEAR, AR(1)
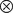
AR(1), and DE
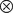
DE. Although, for our particular example, there is no difference in fixed effect (mean model) inference among the models; the covariate p-values for the better fitting LEAR
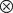
LEAR model are uniformly larger for 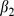 (age), 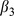 (gender), and 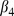 and 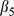 (race). Thus, the results illuminate the difference in fixed effect inference that could occur if there were a covariate of borderline significance. The better fit of the Kronecker product LEAR structure instills more confidence in the results. Therefore, we continue the analysis using the Kronecker product LEAR correlation model.

**Table 3:** AIC values for all combinations of factor specific correlation models.

	Initial Caudate Data Model			Final Caudate Data Model		
	Spatial Model			Spatial Model		
Temporal Model	LEAR	DE	AR(1)	LEAR	DE	AR(1)
LEAR	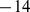 ,298	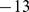 ,903	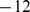 ,717	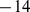 ,307	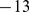 ,912	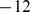 ,722
DE	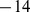 ,295	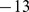 ,900	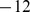 ,714	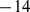 ,304	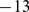 ,909	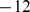 ,719
AR(1)	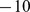 ,377	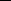 9,983	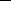 8,768	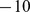 ,386	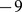 ,992	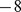 ,774

**Table 4:** Initial full mean model estimates, standard errors, and p-values.

	LEAR 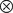 LEAR			AR(1) 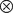 AR(1)			DE 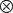 DE		
Parameter	Estimate	SE	P-value	Estimate	SE	P-value	Estimate	SE	P-value
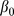	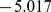	0.046	0.000	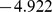	0.030	0.000	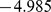	0.044	0.000
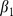	0.004	0.041	0.931	0.001	0.027	0.969	0.003	0.039	0.946
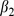	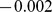	0.003	0.549	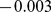	0.002	0.240	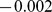	0.003	0.493
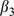	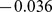	0.038	0.346	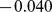	0.025	0.111	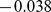	0.037	0.299
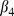	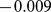	0.034	0.783	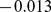	0.022	0.560	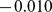	0.033	0.748
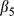	0.016	0.054	0.763	0.014	0.035	0.678	0.016	0.051	0.756

In order to obtain a parsimonious model, the full model defined in equation 9 is reduced via backward selection with 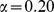. At each reduction step the covariance parameters are re-estimated and the fixed effect covariate with the largest p-value is removed if it is non-significant at the 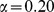 level based on the residual approximation of the 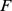-test for a Wald statistic. The final model after reduction is
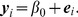
(10)


There is no evidence of a difference in caudate shape between the treated schizophrenics and the controls when taking into account all images taken over time. To ensure that the Kronecker product LEAR correlation model still provides the better fit for the final model, we again model the temporal and spatial factor-specific correlations of the model errors with the continuous-time AR(1), DE, and LEAR structures. **Table 3** contains the AIC values for the final model fits. The Kronecker product LEAR correlation model remains the better correlation structure for the final data model. The BIC corroborates the differences in fits.

**Figure 4:**
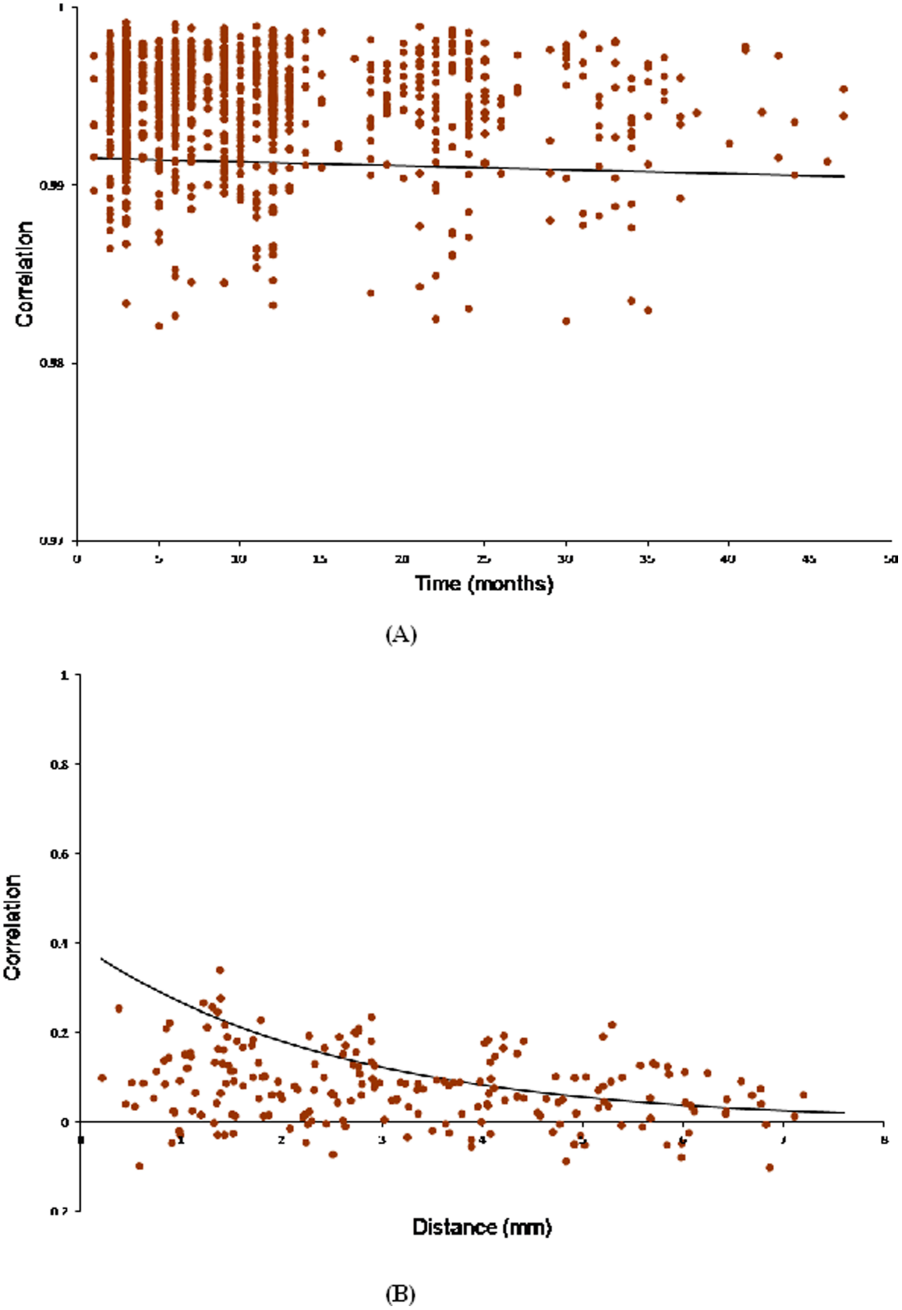
Observed (dots) vs. predicted (curve) correlation. (**A**) as a function of the time between images; (**B**) as a function of the distance between radius locations.

The residual variance estimate and correlation parameter estimates of the Kronecker product LEAR structure (defined in equation 4) for the final data model are given in **Table 5**. Graphical depictions of these estimates are exhibited in **Figures 4A and 4B**, which show the observed vs. predicted correlation patterns as a function of the months between images and millimeters between radii respectively, starting with the minimum temporal and spatial distances for the data. As evidenced by **Figure 4A**, the temporal factor-specific LEAR correlation structure is able to model a correlation function in which the correlation remains high regardless of how far apart in time the images are taken. The fact that the correlation estimates in the time dimension are close to 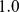
might be considered a problem if the presence of a unit root is expected. This would also present a problem for the competing DE and AR(1) factor-specific models. While much work has been done on the development of unit root tests for time series data [35]–[39], to our knowledge, none are applicable to unbalanced, inconsistently-spaced multivariate repeated measures data modeled with a Kronecker product covariance structure. A test for a unit root might be useful, but developing one is beyond the scope of the present work. The spatial correlations, shown in **Figure 4B**, are modest for radii that are close, and decay slowly toward zero as they become farther apart. The predicted correlation curve appears to slightly overestimate the spatial correlations for small distances. This may be due to the restriction 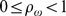, since the model cannot accurately incorporate the negative correlations. One solution may be to add an offset parameter to the model in order to account for negative correlations, i.e.,
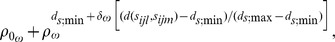
under the condition that

**Table 5:** Final Kronecker product LEAR structure correlation model estimates for caudate data.

Factor	Parameter	Estimate	SE
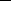	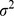	0.405	0.005
Time	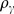	0.992	0.000
	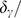 ( 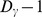 )	0.003	0.001
Space	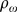	0.381	0.011
	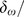 ( 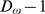 )	0.040	0.004



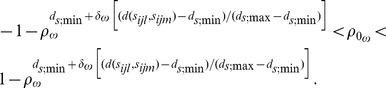



An examination of this approach, and others, will be left for future research.

## Discussion

The Kronecker product LEAR correlation model allows modeling and understanding two factor-specific correlation patterns. Excellent analytic and numerical properties make the structure especially attractive for High Dimensional, Low Sample Size settings that are common in longitudinal medical imaging and various kinds of longitudinal “-omics” data. The structure is able to model a wide variety of correlation patterns with just four parameters. Analysis of the caudate data illustrates the interpretability of the model in a complex context.

An assessment of model fit and inference accuracy in higher dimensional settings is a priority for future research on Kronecker product LEAR correlation models. Also, introducing a nonstationary Kronecker product LEAR correlation or variance structure may prove extremely useful in neuroimaging, since the variability of brain characteristics tends to vary spatially and temporally. Comparing the implementation of the Kronecker product LEAR structure with various modeling and estimation methods will prove valuable. For data that have within-subject correlations induced by three or more factors, as in longitudinal imaging data represented via the m-rep method ([40] has details), the generalization of the Kronecker product LEAR correlation model to 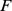 repeated factors would be beneficial.
